# Oxidative Stress as a Potential Mechanism Underlying Membrane Hyperexcitability in Neurodegenerative Diseases

**DOI:** 10.3390/antiox11081511

**Published:** 2022-08-02

**Authors:** Ricardo Pardillo-Díaz, Patricia Pérez-García, Carmen Castro, Pedro Nunez-Abades, Livia Carrascal

**Affiliations:** 1 Biomedical Research and Innovation Institute of Cadiz (INIBICA), 11003 Cadiz, Spain; ricardo.pardillo@gm.uca.es (R.P.-D.); patricia.perezga@gm.uca.es (P.P.-G.); carmen.castro@gm.uca.es (C.C.); 2 Area of Physiology, School of Medicine, University of Cádiz, 11003 Cadiz, Spain; 3 Department of Physiology, Pharmacy School, University of Seville, 41012 Seville, Spain

**Keywords:** oxidative stress, neurodegenerative diseases, hyperexcitability, motor neuron

## Abstract

Neurodegenerative diseases are characterized by gradually progressive, selective loss of anatomically or physiologically related neuronal systems that produce brain damage from which there is no recovery. Despite the differences in clinical manifestations and neuronal vulnerability, the pathological processes appear to be similar, suggesting common neurodegenerative pathways. It is well known that oxidative stress and the production of reactive oxygen radicals plays a key role in neuronal cell damage. It has been proposed that this stress, among other mechanisms, could contribute to neuronal degeneration and might be one of the factors triggering the development of these pathologies. Another common feature in most neurodegenerative diseases is neuron hyperexcitability, an aberrant electrical activity. This review, focusing mainly on primary motor cortex pyramidal neurons, critically evaluates the idea that oxidative stress and inflammation may be involved in neurodegeneration via their capacity to increase membrane excitability.

## 1. Introduction

Degenerative diseases of the central nervous system (CNS) impose substantial medical and public health burdens on populations throughout the world. The overwhelming rise in the prevalence of these disorders parallels the rapid increase in human lifespan. Neurodegenerative diseases, bridging their clinical differences, are characterized by the gradual and irreversible damage and loss of specific neuronal networks. Different brain areas affected will trigger different diseases, with the most common being Alzheimer’s disease (AD), Parkinson’s disease (PD), amyotrophic lateral sclerosis (ALS) and multiple sclerosis (MS) [1]. Among these neurodegenerative diseases AD is the most prevalent, causing between 60% and 80% of dementia cases globally. This disease, derived from an initial deterioration of the hippocampus and a posterior deterioration of the cortex and brainstem regions, is manifested by psychological, motor and specially memory alterations [2]. In the case of PD, the degeneration affects the dopaminergic neurons from the substantia nigra pars compacta, notably producing motor disorders such as bradykinesia, akinesia, stiffness, resting tremor and postural instability as well as non-motor manifestations such as depression, anxiety, sleep disorders, and cognitive and autonomic function alterations [3]. With a lower incidence (2–11 cases per 100,000 people) [4], ALS is the most common disease affecting the motoneurons, and it is triggered by the gradual, irreversible loss of the motoneurons located in the corticospinal tract. Although in its early stages is commonly manifested as muscular weakness, in 25% of patients ALS may be first manifested as bulbar symptoms, characterized by speech and swallowing difficulties. These symptoms constantly evolve until the event of the death of the patient, usually produced by respiratory arrest [5]. Finally, MS is the most prevalent non-traumatic disabling disease among the young adult population. It is an autoimmune inflammatory disease that has been classically attributed to a demyelinating pathology of the CNS. Although optic neuritis is the first symptom of MS for many people, the late stages of this disorder are accompanied with brain and spinal atrophy from which the brainstem and spinal cord syndromes derive [6]. All these neurodegenerative disorders seem to have common etiopathogenic pathways, albeit each of them affect different regions of the CNS, leading to different clinical manifestations. Thus, protein misfolding and aggregation as well as oxidative stress are highly recognizable common features in all these pathologies. In terms of neuronal functionality, hyperexcitability appears to be the common hallmark among all of them and often the degree of excitability of patient’s derived motor neurons correlates with the patient’s rate of survival, especially in ALS [7,8,9]. However, the molecular and cellular mechanisms that lead to the onset of these malfunctions and thus to the development of the disease are complex and still poorly understood. Therefore, an in-depth understanding of the mechanisms underlying the neurodegenerative processes is a necessary step in the development of new treatments capable of preventing the emergence of these diseases or, at least, of delaying their progression [1,10,11]. The aim of this review is to expand the current knowledge on the implications of oxidative stress and hyperexcitability in the pathogenesis and progression of neurodegenerative diseases. We will start by introducing their most relevant aspects and will continue by reviewing the role that hyperexcitability plays in all of them.

## 2. Pathogenesis of Neurodegenerative Disease: Oxidative Stress and Inflammation

**Figure 1:**
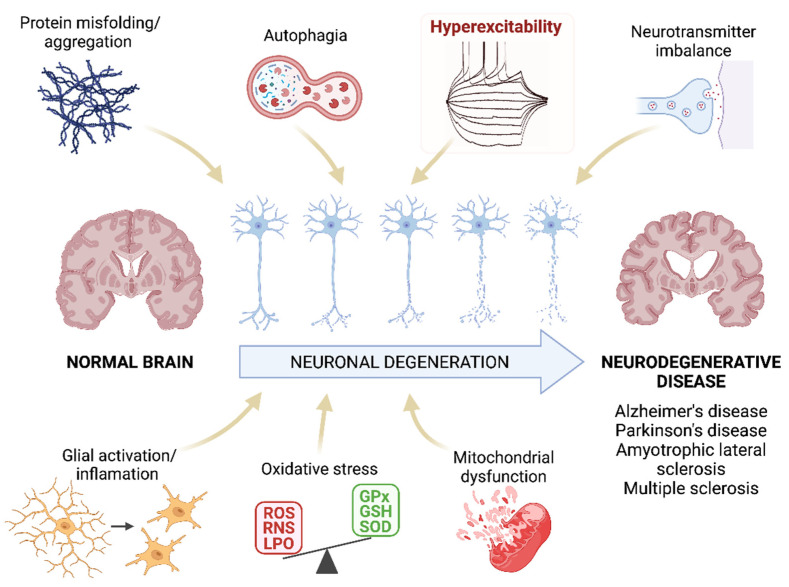
Pathogenesis of neurodegenerative diseases. Schematic representation of the mechanisms that are proposed as the main pathogenic agents of neurodegenerative diseases. We highlight the hyperexcitability as this parameter will be the main focus of this review. ROS: reactive oxygen species; RNS: reactive nitrogen species; LPO: lipid peroxidation; GPx: glutathione peroxidase; GSH: glutathione; SOD: superoxide dismutase. Created with BioRender.com [12].

Irreversible neuronal deterioration is the most common consequence of neurodegenerative diseases -either AD, PD, ALS, MS or others. Although the pathogenesis of these diseases is not fully understood, several neurological components have been already identified as responsible for the neuronal pathology such as increased protein aggregates, glial activation/inflammation, mitochondrial dysfunction, disbalance of neurotransmitters, autophagia or oxidative stress (Figure 1). A comprehensive review of the molecular features and histopathology of all of these pathogenic mechanisms in neurodegenerative diseases is beyond the scope of this review except for oxidative stress but it is worth highlighting several aspects of the role of neuroinflammation because of its interrelation with oxidative stress and hyperexcitability in motor cortex neurons.

### 2.1. Inflammation Triggers Neurodegenerative Diseases

The blood-brain barrier (BBB) is the main protective barrier of the CNS. It has a highly selective permeability and, in addition of selectively allowing the entrance of essential nutrients such as glucose and amino acids, it also regulates the controlled access of immune cells into the brain. An increased BBB permeability to immune cells can disbalance the homeostasis and provoke serious neurological deficits. Thus, a common feature of these CNS diseases is a chronic immune activation, affecting innate and adaptative immune responses. In addition, microglia, astrocytes, endothelial cells, and peripheral immune cells release inflammatory cytokines and chemokines, causing a prolonged proinflammatory state of microglia, increasing the permeability of the BBB and facilitating the infiltration of immune cells into the CNS [13,14].

Microgliosis. Microglia composes the immune system of the CNS. In addition to their phagocytic capacity, active microglia releases proinflammatory molecules such as nitric oxide (NO), tumor necrosis factor α (TNFα), major histocompatibility complex class II (MHC II), prostaglandin-endoperoxide synthase 2 (Cox-2), monocyte chemotaxic protein 1 (MCP-1), and interleukins 1β and 6 (IL-1β and IL-6), playing a crucial role in the inflammatory response. Although microglia is necessary for the elimination of dead neurons, its overactivation is related to a faster progression of neurodegenerative diseases such as AD, PD, ALS and MS [15,16,17,18,19,20]. Under normal conditions, microglia is in a “resting” state morphologically characterized by ramifications that extend and retract so that these cells can constantly monitor the state of the microenvironment that surrounds them. When an abnormality is detected, the resting microglia transitions to an activated state in which the morphology changes to an ameboid shape. Active microglia can be observed, for example, surrounding senile plaques in AD. In addition to activation markers, this microglia expresses proinflammatory mediators such as MCP-1 that contribute to the recruitment of astrocytes in senile plaques [20]. In PD it has been suggested that microgliosis is caused by α-Syn aggregates, and that this active microglia contributes to the death of dopaminergic neurons [21]. Likewise, microgliosis is also a hallmark of neuroinflammation in ALS. Active microglia has been observed by positron emission tomography in the brains of living patients in which, the intensity of activation correlates with the severity of the disease, depicting the role of active microglia in the course of the disease [22]. Microglial activation is also evident in MS, both in early and later stages of the disease. Its activation correlates with axon and oligodendrocyte pathology, in which myelin remnants and cell fragments can be found. In addition to the production of various inflammatory neurotoxic mediators, microglia secretes the proteolytic lipolytic enzymes which ultimately lead to the destruction of myelin [23].

Reactive Astrocytes. Astrocytes are the main components of the glia and perform numerous functions of support and maintenance of the nervous system [24]. Activated astrocytes can give rise to two different forms, known as A1 and A2. While A2 favors tissue repair, A1 favors its degradation. The activation of the latter contributes to neuroinflammation through the release of proteins and inflammatory mediators. Thus, in neurodegenerative diseases astroglia lose its protective function which may cause the collapse of the BBB and neuronal death. For example, in the case of AD, astroglia A1 causes an imbalance in the release of calcium, glutamate and GABA, contributing to the development of the disease [25]. In fact, in many cases the cognitive impairment associated with AD correlates with the number of reactive astrocytes present in senile plaques and with the concentration of proinflammatory mediators produced by them [26]. Moreover, A1 astrocytes promote the formation of amyloid beta (Aβ) plaques [24]. A1 reactivity has also been detected in the substantia nigra of PD patients [27]. At the beginning of the disease, α-Syn inclusions are found inside the astrocytes (also increased in number), which leads to the mobilization of phagocytic microglia and the destruction of cholinergic neurons, producing the clinical symptoms of the disease [28]. Similarly, the degree of astrocytic reactivity correlates directly with the rate of neuronal death in ALS [24,29]. Finally, the existence of reactive astrocytes has also been demonstrated in MS, being the main source of proinflammatory cytokines. These cytokines are the cause of uncontrolled inflammatory reactions with T cell infiltration and associated damage [24,30,31].

Pattern-recognition receptors (PRRs). Innate immune responses are triggered by PRRs. The best known PRRs are the Toll-like receptors (TLRs). It has been proved that CNS cells can activate the innate immune system via motifs related to damage and/or stress, to which TLRs bind. In fact, several studies show that TLRs are upregulated in the brains of patients with neurodegenerative disorders such as AD, PD, ALS or MS [32,33,34]. For example, TLR2, TLR3, and TLR4 are overexpressed in microglia of patients with PD and ALS, activating pathways that lead to the production of proinflammatory cytokines that contribute to neuronal damage [13]. Likewise, the expression of TLR2 and TLR4 is also increased in AD, and it has been shown in vitro that Aβ induces TLR synthesis. However, this increase could also play a beneficial role in neurodegenerative diseases, since TLRs help the absorption and elimination of protein aggregates, including Aβ [35,36,37]. Thus, these receptors seem to play a dual role in the development of neurodegenerative disorders, being able to cause neuronal damage but also favoring tissue regeneration. This suggests that enhancing their beneficial functions may be useful as a therapeutic strategy in the treatment of these pathologies [13].

### 2.2. Role of Oxidative Stress in Neurodegenerative Diseases

**Figure 2:**
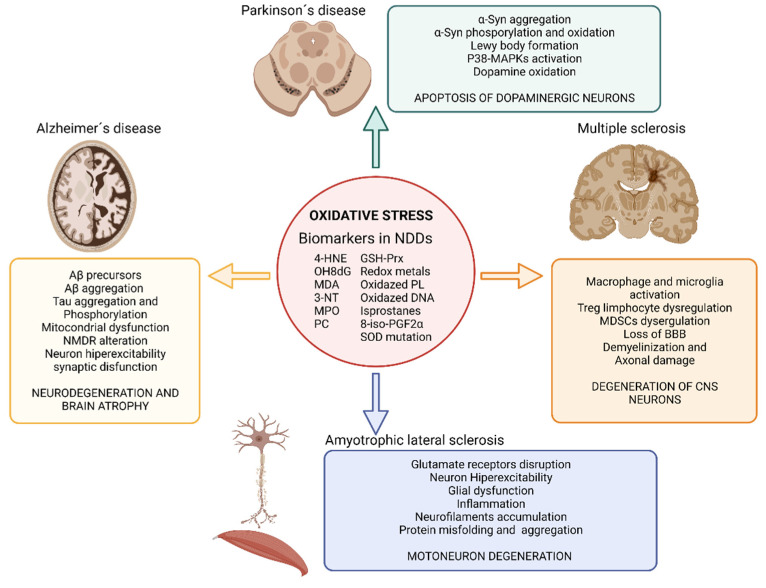
Impact of oxidative stress on neurodegenerative diseases. Illustration showing the impact of oxidative stress on Alzheimer’s disease, Parkinson’s disease, amyotrophic lateral sclerosis and multiple sclerosis and the main biomarkers of oxidative stress found in neurodegenerative disease’s studies. Abbreviatures: BBB, blood brain barrier; GSH-Prx, glutathione-peroxiredoxins; 4-HNE, 4-hidroxinonenal; NDDs, neurodegenerative diseases; NMDR, N-metil-D-aspartato receptors; MDA, malondialdehyde; MAPKs, Mitogen-activated protein kinases; MPO, Myeloperoxidase; 3-NT, 3-nitrotyrosine; OH8dG, 8-hydroxy-2′-deoxyguanosine; PC, protein carbonyl; 8-iso-PGF2α,8-iso-prostaglandin F2α; α-Syn, α-Synuclein; SOD, superoxide dismutase. Created with BioRender.com [12].

Another essential mechanism in neurodegenerative processes is oxidative stress [38]. Oxidative stress is known as the cellular situation in which the free radicals -reactive oxygen and/or nitrogen species (ROS and RNS, respectively)- exceed the natural antioxidant defenses of the organism [39]. The importance of these mechanisms in the development of AD, PD, ALS, and MS lies in the fact that neurons are especially vulnerable to ROS and RNS since they contain high levels of unsaturated lipids susceptible to oxidation and high levels of transition metals that catalyze the synthesis of free radicals [40]. Besides, the CNS has a high metabolic rate and, hence, a high oxygen demand which favors free radical formation. The metabolism of neurotransmitters is also a source of free radicals in the CNS. In addition, the antioxidant defense of the CNS is weaker than in other organs making it even more susceptible to oxidative stress. Under oxidative stress conditions, dysfunctional mitochondria are unable to produce the high energy levels required by neuronal cells to perform their normal biochemical and physiological functions. This mitochondrial impairment makes them vulnerable to rapid cell death [41]. Under physiological concentrations ROS play a crucial role as regulators of various cellular functions such as growth, proliferation, differentiation and survival; they participate in cognitive function and in the maintenance of antioxidant mechanisms [42,43]. However, an excess of ROS and/or RNS can damage cells modifying lipids, proteins, and DNA [38]. ROS produced by mitochondria cause lipid peroxidation (LPO), which alters the cell membrane structure, and integrity affecting cell signaling. In here, we review the evidences that demonstrate the presence of oxidative stress in neurodegenerative diseases and its impact in AD, PD, MS and ALS (Figure 2).

The increased levels of LPO are strongly associated with neurotoxicity in AD [44,45]. Oxidative stress has been detected even before the development of Aβ plaques, thus being proposed as the key player in the etiology of this disease. Oxidative stress contributes to mitochondrial dysfunction, neuronal bodies, and synapsis dysfunctions, as well as to Aβ production [46]. Mitochondrial dysfunction in AD involves impaired mitochondrial complexes that control ATP generation, producing 4-hidroxinonenal (4-HNE) and leading to Aβ accumulation. In addition, increased ROS levels drive to tau aggregates, tau hyperphosphorylation and cell death [38,41]. The imbalance of bioactive metals has also been proposed as one of the mechanisms by which oxidative stress influences AD. Thus, zinc directly affects Aβ precursors [47], and aluminum, zinc, iron, and copper promote Aβ aggregation [48,49]. Similarly, redox-active transition metals could promote Tau phosphorylation [50]. Finally, ROS, amyloid, and Tau protein affect the activity of glutamate receptors and uptake leading to an exacerbate influx of Ca^2+^ in postsynaptic neurons that increase ROS production, oxidative stress, Tau phosphorylation, LPO and hyperexcitability driving to synaptic dysfunction responsible for AD [51,52].

In PD, oxidative stress is associated with α-Syn aggregation and Lewy body formation. Elevated levels of oxidative stress markers such as 4-HNE, protein carbonyl, 8-hydroxy-2′-deoxyguanosine (OH8dG), and 8-hydroxyguanosine have been found in postmortem samples of brains from PD patients. The cascade of events that lead to the degeneration of dopaminergic neurons in PD is also linked to oxidative stress due to the fact that it activates the p38 mitogen activated protein kinase pathway, ultimately leading to apoptosis of the dopaminergic brain cells [53,54]. Furthermore, iron accumulates in the substantia nigra of PD patients enhancing pro-oxidant interactions between iron and dopamine. This imbalance in iron metabolism may also increases the phosphorylation or oxidation of α-Synuclein (α-Syn) in the substantia nigra [55,56]. Iron accumulation in PD patients correlates with the specific spatiotemporal progression of neuronal loss [38,57].

Related to ALS, many studies have reported high levels of oxidative stress during the pathogenesis of the disease. The serum of patients with ALS also shows elevated concentrations of oxidative stress markers such as 3-nitrotyrosine (3-NT), 4-HNE, OH8dG and high activity of the antioxidants defenses such as glutathione peroxidase, superoxide dismutase (SOD), glutathione reductase, and malondialdehyde (MDA) [58,59]. Oxidative stress in ALS has been proposed as a disruptor of glutamate receptors that lead to an increased concentration of glutamate in the synaptic cleft [60,61,62]. This increased glutamate produces motor neurons hyperexcitability and an increment in calcium influx in cells (neurons and glia) as well as mitochondria thus, causing cell damage [63]. ROS also activate glial cells that produce proinflammatory cytokines and more ROS, thus spreading the neurodegeneration toward the whole population [62,64,65]. In addition, ROS has an important impact on the neurocytoskeleton, promoting abnormal accumulation of neurofilaments [66]. Other evidence on the importance of oxidative stress in this disease is the presence of mutations in genes involved in removing ROS. Notably, most of the ALS patients that display the familiar form of the disease (15–20%) present mutations in the SOD1 gene, which plays an important role in the defense against oxidative stress. It has been described more than 150 ALS-related SOD1 gene mutations, affecting various parts of the enzyme. This results in protein misfolding and aggregation, increased ROS production, and redox system disequilibrium, which ultimately leads to nerve cell loss [67]. In sporadic form of ALS, the overexpression of the cystine/glutamate antiporter causes increased levels of ROS and RNS and extracellular glutamate accumulation, which translates into an increase of neuron excitability [41,67].

Oxidative stress is present since the early stages of MS, participating in the demyelination and axonal damage typical of this disease. Postmortem studies demonstrate that in active lesions of the white matter and cerebral cortex, demyelination and neurodegeneration are closely associated with the presence of oxidized lipids (such as oxidized phospholipids and MDA) in myelin membranes and in apoptotic oligodendrocytes and neurons, in which oxidized DNA can be found within their nuclei [68,69,70]. Additional evidence of oxidative stress in MS are the presence of increased protein carbonyls, NO metabolites, SOD, catalase, glutathione reductase, inducible nitric oxide synthase (iNOS), protein carbonyl, 3-NT, isoprostanes, MDA, 8-iso-prostaglandin F2α and products of DNA oxidation in blood, serum, urine and postmortem brain of MS patients [71]. In MS, free radicals trigger an inflammatory response (activating macrophage and microglia) that in turn favors the synthesis of more ROS and RNS, aggravating the problem in a self-reinforcing feedback. The overproduction of free radicals overpasses the antioxidant capacity of the body and produces damage to the nervous tissue [72]. Oxidative stress is also linked in MS to the impairment of regulatory T cells [73] and myeloid-derived suppressor cells (MDSCs) [74] that interfere in the adaptive immune response and in the loss of the BBB [68,75].

## 3. Synaptic Disbalance and Neuron Hyperexcitability in Neurodegenerative Diseases

The spatial organization of neuronal inputs is essential for neural network performance [76]. Various processes, including homeostatic plasticity, regulate the ratio of excitatory and inhibitory (E/I) inputs received by neurons [77]. The balance of this relationship is essential for the correct activity of the different circuits, which implies a correct processing of the information in order to generate an adequate response. According to Lopatina et al., (2019) “E/I balance controls synaptic inputs to prevent inappropriate response of neurons to input force and to provide the same plasticity regimes for all synapses within the neuronal population”. Therefore, it seems reasonable to think that different events that could cause an imbalance in the E/I ratio may be the basis of various pathologies of the CNS, including several neurodegenerative diseases [78,79,80].

### 3.1. Hyperexcitability in AD

Cortical and hippocampal network hyperexcitabilities are characteristic in patients with AD. Both cognitive function and behavioral patterns are intrinsically regulated by neuronal excitability. For this reason, the increase in excitability observed from the early stages of AD has been proposed as one of the detonating contributors to memory loss in this disease [81,82]. Hyperexcitability of the cerebral cortex may be manifested as both clinical and subclinical epileptic seizures. In fact, while the formers are suffered by the 10–20% of Alzheimer’s patients, the latter occur in up to 40% of cases [83,84,85]. Regarding hippocampal activity, the hypothesis of the inverted U-shaped trend is becoming popular among the scientific community. Several studies with AD patients show that before and during the early stages of the disease, when memory loss is still mild or not evident, neuronal activity is intensified in the hippocampal, parahippocampal and fusiform regions when individuals perform a memory-encoding task. As the disease progresses and cognitive decline and memory loss become evident, activity decreases to a pronounced hippocampal and entorhinal cortex hypoactivity [85,86,87]. Also, in cell and animal models of AD, abnormal neuron excitability has been reported in the cortex and hippocampus, with neurons showing a higher frequency of action potential (AP) firing, as well as a lower recruitment threshold to fire. However, this hyperexcitability, in animal models of AD, manifests at different stages of the disease, depending on the area of the brain and the animal model used. Thus, in the double transgenic mice model of AD (APP23xPS45), a study using two-photon Ca^2+^ imaging to analyze brain activity demonstrated a progressive cortical hyperactivity without the shift from hyper to hypoexcitability shown in patient studies [88]. Nevertheless, another study performed in the APP23xPS45 mice suggest that in the hippocampus hyperxcitability starts early and decreases with age, while hypoactivity increases over time, as shown in clinical studies [89,90].

Although the causes of hyperexcitability are not yet clearly understood, it seems that several factors, such as, Aβ proteins and tau, glutamate and inhibitory interneurons could be involved [91]. Thus, increased neuronal excitability is observed in neurons closed to Aβ plaques [90]. Some studies proposed that Aβ plaques disrupts voltage-gated ion channels responsible for maintaining neuronal membrane potential or calcium homeostasis [92]. Moreover, in animal models of AD soluble forms of Aβ produce alterations in the E/I ratio at the neuronal level and it has been proposed that this imbalance can be restored by treatment with benzodiazepines, which pharmacologically enhance GABAergic inhibition and prevent cellular hyperexcitability, thus ameliorating memory deficits [89]. It is worth mentioning that the influence of Aβ on neuronal activity is reciprocal, since the repair of the E/I balance can prevent the formation of Aβ plaques. According to Zott et al., 2018 “Aβ-dependent impairments of synaptic inhibition can account for many of the known defects in the AD brain, including impairments of gamma and slow oscillations, epileptic activity and seizures, and hippocampal hyperactivation, as well as cognitive impairment”. Furthermore, in AD patients and animal models, it has been found a reduction in the expression of excitatory amino acid transporters 1–2 (EAAT1-2) [93] and glutamine synthetase [94] increasing the availability of glutamate that could lead to the hyperexcitability. Additionally, other mechanisms that mediate neuronal hyperexcitability could be implicated, such as changes in voltage-dependent sodium channels (Nav), especially Nav1.6 since they are mainly expressed in excitatory pyramidal neurons and therefore directly modulate the excitability [85,95]. Nav1.6 channels are also closely related to the Aβ protein, and it has been reported that in AD patients, Nav1.6 channels colocalize with Aβ increasing the firing rate through these channels and, therefore, conducing to hippocampal hyperexcitability [95]. These channels have been shown to increase BACE1 transcription in the APP/PS1 mouse model of AD, being BACE1 the limiting enzyme in the Aβ production. Furthermore, in Nav1.6-knockdown mice the Aβ deposition decreases, and this alleviates the damage on synaptic plasticity and cognitive function [96]. Finally, and among other factors, a decrease of inhibitory neuron function has been linked to hyperexcitability in AD For example, in the APOE murine model of AD a reduction in the responsiveness of excitatory neurons to GABAergic inhibitory inputs has been observed [87]. 

Hyperexcitability in AD has been considered for a long time a compensatory mechanism to reduce the effect of neurodegeneration and synaptic deficit. However, the mixed quality evidences found so far do not support the hypothesis due to the great number of data demonstrating that this phenomenon occurs in the early stages of the disease, even before the significant neuronal loss. This indicates that it should be considered a trigger factor of the disease rather than a compensatory mechanism.

### 3.2. Hyperexcitability in MS

Although synaptic alterations and neuronal hyperexcitability have not been as extensively studied in MS as in the case of AD, these factors are increasingly being proposed as key points in the early degeneration that occurs in this disease. As an example to emphasize its importance, animal models of MS show an increase in excitatory glutamate and a decrease in inhibitory GABA transmissions [97,98,99]. In addition, in cerebrospinal fluid and brains of MS patients both glutamate and GABA levels are altered, as well as glutamate clearance and glutamate receptor expression. All these alterations result in an E/I imbalance, promoting hyperexcitability and cortical damage. Moreover, cortical hyperexcitability has been associated with imbalances in the concentrations of proinflammatory and anti-inflammatory molecules in the cerebrospinal fluid, suggesting that hyperexcitability could lead to inflammatory synaptopathy being a critical mechanism in the development of the pathology [100].

### 3.3. Hyperexcitability in ALS

In ALS patients, as well as in AD patients, neuronal hyperexcitability appears to precede the onset of clinical symptoms of the disease. Cortical hyperexcitability begins even before neuromotor degeneration. As the disease progresses, it becomes more exacerbated in ALS patients who also suffer cognitive impairment [101,102]. Clinically, fasciculations, hyper-reflexia and spasticity are manifestations of this cortical hyperexcitability, which may be linked to both increased facilitation and disinhibition in the primary motor cortex [103]. Cortical hyperexcitability is reflected in the decrease in motor thresholds, since they are proportional to the density of motoneurons present in the corticospinal and corticobulbar tracts [104]. Although resting motor threshold may be an indicative of the intrinsic excitability of corticospinal motor neurons, it should be noted that it is also influenced by glutamatergic and GABAergic signals from the motor cortex [105,106]. The reduction in motor thresholds occurs mainly at the beginning of the disease. It is associated with profuse fasciculations and hyper-reflexia and it contributes to the spread of the disease [9,107]. However, some studies have shown that the progression of the disease correlates with a gradual increase in motor thresholds, which implies inexcitability of the motor cortex, following the U-inverted trend seen in AD [106,108].

Although it has been reported that early cortical hyperexcitability could be a compensatory mechanism in response to spinal motoneuron degeneration [109], molecular studies support the relevance of cortical hyperexcitability in the development and progression of ALS. At the molecular level, cortical hyperexcitability seems to be linked to glutamate excitotoxicity. Supporting this idea, a downregulation of the astrocytic glutamate transporter EAAT2 occurs pre-clinically in ALS, resulting in an increase in glutamate activity. Besides, the activation of the EAAT2 transporter inhibitor, caspase-1, has been documented in the transgenic SOD-1 mouse prior to cell death [110,111]. On the other hand, several studies evidence that this cortical hyperexcitability is related to the impairment of local inhibitory networks, corroborated by the existence of a GABA reduction in the primary motor cortex of these patients [112] and by reduced mRNA expression of GABA_A_ receptor subunits [113]. Changes in morphological and electrophysiological features of cortical neurons also occur in ALS models and they result in neuron hyperexcitability [114,115]. In the wobbler mouse, a mouse model of ALS, pyramidal neurons from layer V present hyperexcitability characterized by a lower current threshold for AP and a higher input resistance. Phasic and tonic GABAergic inhibition is reduced in the wobbler mouse explaining this phenomenon [114]. Synaptic activity of layer V pyramidal neurons from the motor cortex was also studied in both SOD-1 and transactive response DNA binding protein 43 (TDP-43) mice. In these models it has been demonstrated an increased excitatory synaptic neurotransmission resulting in decreased apical and basal dendritic spine densities without compensatory changes in synaptic inhibition [116,117]. Voltage–gated Kv1 channels (KCNA1) are widely expressed on apical dendrites and serve as inward-rectifier currents preventing neuronal hyperexcitability. Therefore, the decrease in density of KCNA1 channels support the existing hyperexcitability in SOD-1 and TDP-43 mice [116]. On the other hand, cortical neurons of SOD-1 mice show an increase in persistent sodium currents, as seen in ALS patients, implying a relationship between these currents and the increased neural excitability [115,118]. The recognition of cortical hyperexcitability as a premature and specific characteristic of ALS suggest its potential utility as a diagnostic early biomarker of this pathology [107].

Although there is a general consensus on the presence of hyperexcitability in the early stages of neurodegenerative diseases, the exact moment in which it appears, why it appears, or how it can lead to reduced neuronal activity require future studies.

## 4. Impact of Oxidative Stress in Motor Cortex Neurons Hyperexcitability

As we have reviewed above, hyperexcitability is a hallmark of most degenerative diseases, however, whether this hyperexcitability is directly linked to inflammation, aggregated proteins or oxidative stress still needs to be fully understood. In this section we will review the effect of oxidative stress upon neurons hyperexcitability focusing specially on the direct effect of this mechanism in pyramidal neurons from the primary motor cortex, which are selectively degenerated in ALS.

### 4.1. Neuronal Excitability and Oxidative Stress

**Figure 3:**
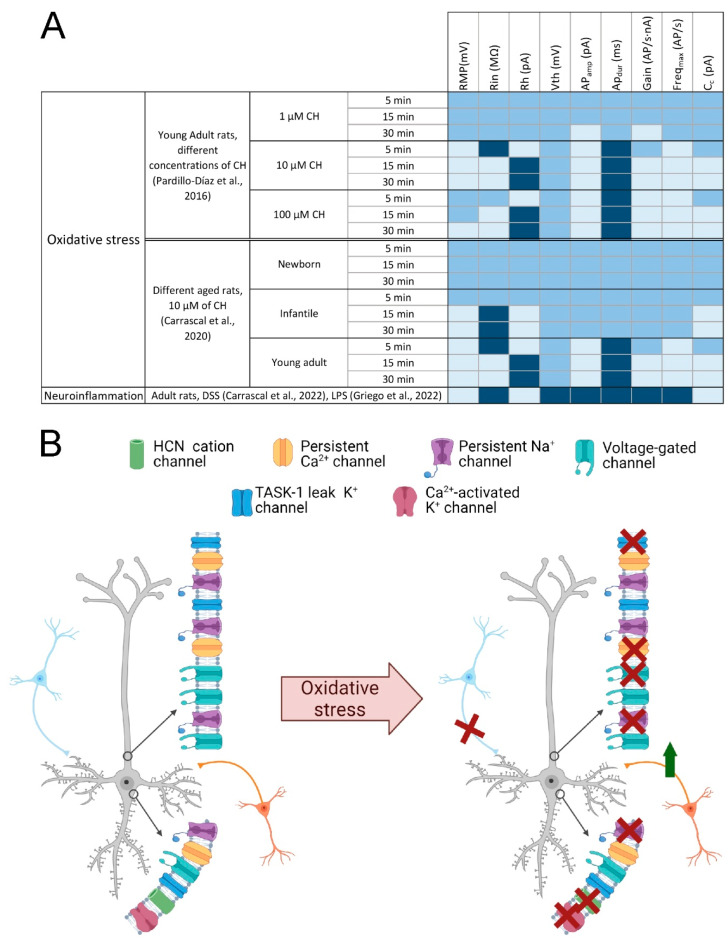
Involvement of oxidative stress in neuronal damage. (**A**) Table summarizing the main changes evoked by oxidative stress and neuroinflammation on motor cortex neurons membrane properties. The electrophysiological parameters that increase under these conditions are represented in dark blue, the parameters that remain similar to control in medium blue and those that decrease in light blue. (**B**) Alterations of ion channels due to oxidative stress. Schematic drawings of two neurons, one unaffected by oxidative stress (**left**) and one affected (**right**); red crosses indicate loss of ion channel activity or loss of synaptic input; the green arrow indicates the increase in synaptic inputs; blue neurons represent GABAergic inputs, orange neurons represent glutamatergic inputs. Abbreviatures: AP_amp_ and AP_dur_, action potential amplitude and duration; C_c_, cancellation current; CH, cumene hydroperoxide; DSS, dextran sulphate sodium; Freq_max_, Maximal Frequency; HCN, hyperpolarization-activated cyclic nucleotide–gated; LPS, lipopolysaccharide; Rh, rheobase; Rin, input resistance; RMP, resting membrane potential; TASK, TWIK-related acid-sensitive K^+^; Vth, voltage threshold. Created with BioRender.com [12].

To verify the effects of oxidative stress on neuronal excitability and on the electrophysiological properties of pyramidal neurons, several in vitro studies have been carried out by our group using patch-clamp whole-cell techniques in young adult rats. In order to induce the formation of free oxygen radicals and thus cause oxidative stress, we used Cumene Hydroperoxide (CH) [119]. CH, and thus oxidative stress, produce many changes in the electrophysiological properties, both active and passive, of pyramidal neurons [120] (Figure 3A). Regarding the passive properties, we observed that the resting membrane potential progressively depolarized. This depolarization may be due to the activity inhibition of the K^+^ channels, as we can see in the work of Nakaya et al. (1992) [121] (Figure 3B). Another mechanism by which this depolarization can occur is the ROS-mediated blocking of TWIK-related acid-sensitive K+ (TASK) channels 1 [122] (Figure 3B). On the other hand, the membrane resistance increased to later decrease below control values, showing a biphasic behavior. The increase observed in resistance may be linked to the inhibition caused by oxidative stress on inward rectifier K^+^ channels, TASK-1 leak K^+^ channels, persistent Na^+^ and Ca^2+^ channels and the Ih channels [122,123]. Moreover, the observed decrease in resistance may be due to the LPO produced by CH. As mentioned above, lipids are the basic structural components of the cell membrane and this LPO may alter its structure causing changes in membrane permeability [119]. As the membrane had an ohmic behavior, the rheobase showed compensatory changes in response to changes in resistance [120]. Looking at the active properties, we observed that the amplitude of the AP decreased while its duration increased. These results coincide with those observed by Jovanovic and Jovanovic in their study with leech neurons [124]. This alteration in the amplitude of the AP may be due to various changes produced by oxidative stress on different conductances. Jovanovic and Jovanovic (2013) together with Nakaya et al. (1992) advocate a blockade of Ca^2+^-activate K^+^ channels [121,124]. For their part, Angelova and Müller (2005) postulate that the conductances altered by oxidative stress are those that are involved in repolarization, such as the voltage-dependent transient current or the delayed rectifier K^+^ current [125]. In our studies, oxidative stress also affected repetitive firing properties. Most of the neurons studied maintain these repetitive properties, but changes in their maximum frequency and gain were noted since these parameters were diminished. In some of the cells even the ability to fire repetitively was abolished. These results also coincide with those seen by Jovanovic and Jovanovic, (2013) [124] but contrast with those obtained by Nani et al., (2010) [122]. The decrease in neuronal excitability may be due to the depression of persistent Na^+^ conductances [126]. The loss of the ability to fire repetitively may be related to the inhibition of fast Na^+^ currents [127]. Alterations in the firing of these neurons may be due to changes in the probability of Na^+^ channel opening, as demonstrated by Huang et al., (2009) when they found that H_2_O_2_ affects the probability of opening of voltage-gated channels by oxidizing the cysteine residues of the protein monomers that conform the channel [128]. In addition to these changes, we also observed a decrease in the working range of the pyramidal neurons since the cancellation of the repetitive properties occurred at lower current intensities as the exposure time to CH increased [120].

### 4.2. Neuronal Excitability and Time and Dose Dependent Effects of Oxidative Stress

In our previous studies, oxidative stress affected the membrane properties of pyramidal neurons in a dose-dependent manner [129]. To study the changes that occur, we subjected the pyramidal neurons to increasing concentrations of CH (1, 10 and 100 μM) for 30 min. The results obtained showed a large number of changes produced in a dose and time dependent manner (Figure 3A). When cells were subjected to a small concentration of CH (1 µM) some changes in both passive and active properties were observed. In the passive properties, a blockade of the inward rectifications (sag) was observed but only with a long exposure [130]. Regarding the active properties, a decrease in the amplitude of the AP and the gain was found. These effects may be, as we have commented previously, the consequence of a blockade of the conductances that intervene in the repolarization of the AP and the Na^+^ ionic gradients [120,121,124,125]. When a concentration of CH of 10 µM is used, a depression of the excitability of the pyramidal neurons of the motor cortex is produced, characterized by a decrease in membrane resistance, the amplitude of the AP, the gain and the maximum firing frequency [129]. Finally, when a high concentration of CH (100 µM) was used, the depression in the excitability of these neurons was even greater because the ability of these cells to produce repetitive discharge of Aps is completely blocked. These results are in agreement with those obtained by Jovanovic and Jovanovic (2013) but contrary to those observed by Nani et al., (2010). The reason for this discrepancy may be because H_2_O_2_ can only perform similar effects to CH when used at a concentration 10 times higher [131].

As we have commented previously, the changes observed are dependent on concentration and time. Thus, when high concentrations of CH or long exposure times were used in our experiments, the working range narrowed. This decrease in the working range was characterized by an increase in the rheobase and a reduction in the cancellation current [129,132]. All the effects caused by oxidative stress were practically irreversible. However, by using melatonin, due to its antioxidant characteristics [133], LPO was prevented and with it the changes that occurred in the electrophysiological properties of pyramidal neurons.

### 4.3. Oxidative Stress, Synaptic Depression and Hyperexcitability

Our previous studies also showed that oxidative stress affects the synaptic events of the pyramidal neurons of the motor cortex, causing synaptic depression. These alterations occurred in a time-dependent manner [134]. CH affected spontaneous postsynaptic conductances (PSCs), whether they were excitatory (EPSCs) or inhibitory (IPSCs), causing their frequency, amplitude and decay rate to be lower. We can find similar results in the study by Nani et al., (2010) [122] where they observed a reduction in the frequency of spontaneous synaptic events due to oxidative stress caused by hydrogen hydroperoxide. However, it had no effect on mEPSCs or mIPSCs. These alterations caused by CH may be due to presynaptic mechanisms that cause a decrease in the release of neurotransmitters linked to APs (in AP-dependent neurotransmitter release). In summary, based on our previous work, we can assume that the functional alterations caused by CH are due to pre- and postsynaptic mechanisms. Furthermore, CH induced a postsynaptic inward current that underlined a membrane depolarization [134]. As there was a decrease in the input flow of the inhibitory premotor interneurons, that is, a reduction in GABAergic inhibition, a transient hyperexcitability occurred, characterized by an increase in resistance and a decrease in rheobase.

This work showed that this depression is already observable on GABAergic synaptic transmission after 5 min application of the CH and this correlates with the transient increase in input resistance, found in current clamp experiments [120,134]. Based on these works it may be suggested that the overall effect of LPO induced by CH was to depress the input flow from the premotor interneurons that promote an unexpected transient hyperexcitability onto pyramidal neurons of the motor cortex (Figure 3B). This hyperexcitability is similar to that observed by some authors such as Nieto-Gonzalez et al., (2011) [113], Maekawa et al. (2004) [135] or Zanette et al. (2002) [109] in their work with wobbler mice, which showed an increased input resistance and excitability by lack of GABA_A_ receptor-mediated influences.

### 4.4. Temporal Course of Neuron Hyperexcitability

Throughout lifetime there is an increase in oxidative stress physiologically as the body loses the ability to maintain the balance between oxidant and antioxidant substances [136]. To see how oxidative stress alters the properties of the pyramidal neurons of the primary motor cortex, we developed a study in which we used rats classified into three experimental groups: Newborn (P2–P7); infantile (P11–P15); and young adult (P20–P40). As in previous studies, we used CH (10 µM) to induce oxidative stress in our population and performed electrophysiological recordings using the whole-cell patch-clamp technique for 30 min. When analyzing the data of the newborn group, we observed that there were no significant alterations in any of the parameters analyzed, the excitability of the membrane did not vary. On the other hand, in the infantile group we did observe some changes. The resting membrane potential depolarizes, the input resistance increases, and the rheobase decreases. These effects were derived from the blocking of a tonic GABAergic conductance. Greater alterations in membrane properties were observed in a previous study on young adult rats by Pardillo-Díaz et al. (2015) [120], suggesting that vulnerability to oxidative stress increases with age.

As we have commented previously, CH causes LPO that will affect the lipids that compose the membrane of pyramidal neurons. To make an indirect measurement of this peroxidation, we quantified the concentration of protein-bound 4-HNE in the brain slices of the three experimental groups (newborn, infantile and young adult) because 4-HNE is a product of LPO, which modifies proteins reacting with an amino group or with a protein side chain, thus leading to protein damage [137]. The result was a significant increase in 4-HNE in the homogenates of infantile and young adult rats. However, in the homogenates of the newborns, no increase in 4-HNE was observed, showing a greater capacity of younger animals to resist or respond to oxidative stress. To study this resistance to oxidative stress, measurements of the content of glutathione (GSH) and free thiols were made. GSH is the most abundant non-protein free thiol found in cells and plays a significant role in various endogenous pathways to fight ROS species and resist conditions of oxidative stress [138]. We observed that the group of newborns presented a formidable increase at this levels. This correlated with the time-dependent increase in glutathione reductase activity, indicating that newborn rats have a greater buffering capacity to resist oxidative stress. Consistent with our study, Deepashree et al., (2019) [138] observed an age-related decline in antioxidant defenses following ethanol-induced oxidative stress on *Drosophila* and Guevara et al., (2011) [139] detected a significant age-related reduction in antioxidant enzyme activities, including SOD and catalase, in rat brains.

Since these results show that the pyramidal neurons of newborn rats increase their GSH in response to oxidative stress and that these are the ones with the greatest resistance to oxidative stress, we developed a study to demonstrate that GSH is able to prevent alterations in the cellular membrane. To do this, brain slices from infantile rats were pre-treated with 4 mM of glutathione monoethyl ester (GSH-MEE) to prevent the alterations produced when 10 µM of CH was applied. No changes were observed after 30 min of exposure to 10 µM CH in any membrane property, either active or passive, in the presence of 4 mM GSH-MEE, which indicates that this GSH-MEE is acting as a neuroprotector.

Therefore, based on all our previous results, it is reasonable to hypothesize that damage caused by oxidants such as CH mainly contributes to neuronal dysfunction in aged brains, and therefore, neurodegenerative diseases such as ALS are more likely to occur in the older population [140].

### 4.5. Peripheral Inflammation, Neuroinflammation and Hyperexcitability

Neuroinflammation is a pathological process that is involved in the appearance of certain neurodegenerative diseases such as PD, AD or ALS [19]. During this neuroinflammation, activated microglia releases inflammatory mediators and ROS such as NO. This NO comes mainly from an increase in the expression of iNOS, or in the form that only appears in neurons known as nNOS [141,142].

There are certain diseases that cause peripheral inflammation, known as inflammatory bowel diseases, among which we can find ulcerative colitis, and that can cause inflammation of the CNS [143,144,145]. To verify this fact, an experiment was carried out in which a group of rats was exposed for 7 days to 3% dextran sulphate sodium (DSS). Once these animals developed an acute colon inflammation, it was found that this peripherical inflammation was capable to induce inflammation in the CNS analyzing the activation of microglia and astrocytes in the primary motor cortex. Treatment with DSS produced an activation of the microglia as there was an increase in the size of these cells and in the number of iNOS positive cells. There were even iNOS-positive cells that were not microglial cells, which means that both glial and non-glial cells reacted to peripheral inflammation by increasing iNOS expression. In turn, astrocytes were also activated by DSS treatment since an increase in glial fibrillary acidic protein (GFAP) expression was seen compared to the control situation. Some works suggest that high levels of NO may induce hyperexcitability and kill neurons through inhibition of K^+^ resting currents and activation of transient receptor potential channels, both leading to neuronal depolarization while others found that NO increased glutamate release and affected the voltage- and/or ligand-gated ion channels [146,147]. With these results we can say that the acute inflammation of the colon induced by treatment with DSS causes neuroinflammation and activation of microglia and astrocytes of the primary motor cortex [148].

From the point of view of membrane excitability of pyramidal neurons, DSS treatment affects the electrophysiological properties of these cells. Regarding the passive properties, the resting membrane potential is more depolarized than in the control condition and the membrane resistance increases considerably in the treated animals. When analyzing the active properties, we see that the rheobase is reduced in relation to the increase in resistance. The duration and amplitude of the AP are not altered, but the repetitive firing properties are, since we see that there is an increase in the gain and a decrease in the cancellation current in the treated cells. All this indicates that neuroinflammation induced by DSS-treatment causes a state of hyperexcitability in the pyramidal neurons of the primary motor cortex and motor deficits [148].

As stated, the activation of the immune system can cause different changes in the conductance of nerve cells that can lead to a state of hyperexcitability. From this starting point, Griego et al., (2022) also developed a study in which they have studied the changes that occur in the electrophysiological properties of the pyramidal neurons of the prelimbic cortex [149]. By injecting 1 mg/kg of intraperitoneal lipopolysaccharide (LPS), the activation of the immune system is triggered and 24 h later they have studied the changes that occur in the properties of these cells [150]. The modulation of ion channels underlying passive and active membrane conductances is a critical element for expressing potentiation of intrinsic excitability [151,152]. In this study, neurons treated with LPS show increased activity of Na^+^ (INa) currents as the functionality of Na^+^ channels increases. LPS causes changes in fast K^+^ currents resulting in hyperexcitability. Analyzing the passive electrophysiological properties, the cells treated with LPS also showed an increase in resistance and time constant and a decrease in rheobase that corresponds to the variation in resistance values as previously explained [120,149]. Regarding the active properties, they have observed that the administration of LPS alters the AP characteristics: reduces the amplitude of the AP, increases the AP firing rate, and impairs the firing pattern, even eliminating the ability of these cells to fire repetitively. Another altered parameter, which is closely related to hyperexcitability is the afterhyperpolarization (AHP) produced by calcium-activated potassium currents. In cells treated with LPS, the AHP is reduced, favoring an increase in the firing rate of these cells. In short, the alteration of the sodium and potassium currents (specifically the fast ones) and of numerous channels such as the calcium-dependent K^+^ channels, cause alterations in the passive and active electrophysiological properties of these cells, causing a state of hyperexcitability. In conclusion, the results obtained from studies inducing peripheral inflammation or induced immune activation, are quite similar to those obtained by our group inducing oxidative stress in experiments carried out in in vitro preparations [120,129].

## 5. Conclusions

We have reviewed in here that neuroinflammation, oxidative stress, and neuronal hyperexcitability are closely interconnected factors that lead to the generation and progression of neurodegenerative diseases, including AD, PD, ALS, and MS. Aβ and tau, α-Syn. TDP-43 and SOD-1 are the proteins involved in AD, PD and ALS, respectively, forming the characteristic protein aggregates of each disease. They establish a crosstalk with microglia and astrocytes, triggering neuroinflammation and promoting ROS formation being associated in some cases with neuronal hyperexcitability. Oxidative stress is the result of the imbalance between the production of free radicals and the body’s antioxidant defenses, constituting another key point in the pathogenesis of neurodegenerative diseases. The production of ROS implies the existence of mitochondrial dysfunction and therefore, failures in energy production. In addition, ROS cause protein and DNA damage, as well as changes in cell membrane structure through LPO, affecting signaling pathways and neuronal electrophysiological properties that lead to increased neuronal excitability in the early stages of neurodegenerative diseases. This results in cell exhaustion, with consequent neuronal hypoactivity in later stages of the disease ending in neuronal death. Previous studies from our group show that oxidative stress generated by the administration of CH produces almost irreversible alterations in the electrophysiological properties, both active and passive, of the pyramidal neurons of the primary motor cortex. Age-dependent alterations in the redox buffering capacity of the neurons, make neurons susceptible to ROS leading to alterations that cause functional changes in the cells and that compromise the excitability of the neurons. All these effects lead to the neuronal death that characterizes neurodegenerative diseases. Interestingly, we discuss that a partial protection of these effects might be achieved with the use of naturally occurring antioxidants such as melatonin.

It is necessary to continue advancing in the study of the interconnections between neurodegenerative disorders, hyperexcitability, oxidative stress and inflammation since, if demonstrated, an early administration of antioxidant substances could represent a preventive treatment to delay the pathogenesis of these devastating human diseases.

## Data Availability

Data is contained within the article.
